# High Flow Priapism in a Pediatric Patient after Circumcision with Dorsal Penile Nerve Block

**DOI:** 10.1155/2016/6976439

**Published:** 2016-08-28

**Authors:** Michael A. Granieri, Joseph J. Fantony, Jonathan C. Routh

**Affiliations:** Department of Surgery, Division of Urology, Duke University Medical Center, Durham, NC 27705, USA

## Abstract

We report the first documented case of high flow priapism after circumcision with dorsal penile nerve block. A 7-year-old male who had undergone circumcision three years before presented to our institution with a 3-year history of persistent nonpainful erections. Workup revealed a high flow priapism and, after discussion of the management options, the patient's family elected continued observation.

## 1. Introduction

Circumcision is one of the most common surgical procedures in the world. An estimated 25–33% of the world male population is circumcised, with rates as high as 70% in the United States [1].

Neonatal circumcisions are commonly performed under local anesthesia while older children routinely have a combination of general and local anesthesia. The dorsal penile nerve block (DPNB) is a safe and appropriate anesthesia technique aimed at delivering a local anesthetic agent at a dose of 1 mL + 0.1 mL/kg body weight around the main trunk of the dorsal nerve [2]. Care is taken to avoid the midline where dorsal vessels course. A myriad of complications from circumcision have been reported including bleeding, phimosis, skin bridging, infection, and urethrocutaneous fistula [3, 4]. To date, we are unaware of any reports of high flow priapism after circumcision with DPNB.

High flow priapism is an extremely rare condition in the pediatric population with an unknown true incidence. The most common cause is perineal trauma creating an arteriocavernosal fistula; however, there have been reports of idiopathic high flow priapism [5].

In this case report, we present the first documented case of high flow priapism in a child after circumcision with a DPNB.

## 2. Case History

A 7-year-old African American boy presented to our institutional Emergency Department (ED) in February of 2016 with a three-year history of persistent, nonpainful erections. Approximately three years before the patient had a circumcision and frenulotomy for balanitis. A week after surgery the patient's mother noted intermittent erections that persisted until his one-month follow-up visit. He was examined at this visit and the presence of a persistent nonpainful erection consistent with high flow priapism was documented. Due to the reported intermittent, nonpainful nature of his erections, the patient was scheduled to return in one month for reevaluation. A referral to pediatric hematology was also made. The patient did not present to either appointment and was lost to follow-up. Three years later, he was evaluated by a local urologist for nocturnal enuresis. After his physical exam, the patient was promptly referred to our institution for urgent evaluation.

Discussion with the mother and grandmother in the ED revealed a three-year history of persistent, nonpainful erections that began shortly after circumcision. The patient's mother had not seen her son flaccid at any point during this time period. There was no family history of sickle cell trait or disease, and the family and patient denied any constitutional symptoms or perineal/genital trauma. The patient was voiding without difficulty.

**Figure 1:**
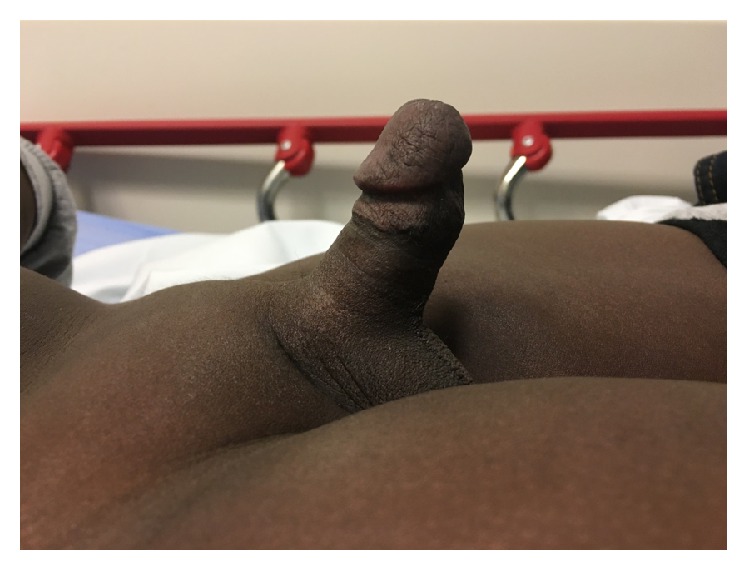
Physical examination during ED visit.

Physical examination revealed a fully erect, nontender penis with rigid corpora cavernosa, a soft glans, and mild dorsal curvature (Figure 1). There were no signs of perineal or genital trauma. The initial workup for priapism was performed and returned with normal values for his CBC, urine toxicology, peripheral smear, and reticulocyte count.

A cavernosal blood gas analysis was collected with the following values: pH 7.45, pO_2_ 193 mmHg, and pCO_2_ 33 mmHg.

He was observed in the ED and was noted to have a persistent erection. After discussion with the patient's mother, he was discharged to home with follow-up in one week to discuss long term management. In the interim, he underwent a penile color Doppler study, which showed no sign of arteriocavernosal fistula.

At follow-up a detailed discussion with the mother about the treatment options was performed. A formal recommendation of angiography with superselective embolization by interventional radiology was made. The patient's family declined any intervention and elected to return in one year for follow-up.

## 3. Discussion

High flow priapism in the pediatric population is primarily due to an arteriocavernosal fistula and leads to persistent nonpainful erections. Prior case reports have identified sickle cell anemia, leukemia, Fabry's disease, or sildenafil toxicity as potential etiologies [6–10].

In our case, the temporal relationship of the onset of priapism and circumcision with no other obvious etiology leads us to believe that an arteriocavernosal fistula was most likely created by the DPNB. To date, this is the first reported case of high flow priapism caused by a DPNB during circumcision. This is even more remarkable since circumcision is one of the most common procedures performed in the world.

Interestingly, the patient's penile color Doppler ultrasound was negative for arteriocavernosal fistula. In an adult population, the sensitivity of this exam nears 100% for detecting these fistulae, and its performance actually improves over time [11]. Given that his iatrogenic injury occurred three years before, we would expect this test to be likely to diagnose the fistula if it were present. However, the studies evaluating the performance characteristics of the penile color Doppler ultrasound do not include a strictly pediatric population, making their applicability to our case somewhat limited. The radiologist who performed this exam anecdotally stated they perform this test rarely and do not consider this negative result to be definitive proof of the absence of fistula.

Despite the negative imaging findings, the patient's clinical history and objective data at the time of the ED visit are clearly consistent with high flow priapism, and thus we will manage it as such. Further imaging with MRI or angiography was not pursued as the parents expressed no interest in treating the problem at this time due to concern of long term erectile dysfunction.

The treatment options for high flow priapism include observation or intervention with superselective arterial embolization or surgical ligation of the offending vessel [12]. Watchful waiting is usually the first line management of high flow priapism [13]. Should this fail, percutaneous transcatheter embolization is usually the most effective treatment [14]. In our case, the patient has been observed for 3 years with no resolution. Although one cannot predict the timing of spontaneous resolution, a series by Corbetta et al. reported spontaneous resolution of high flow priapism in three patients in 14, 27, and 36 days after initial insult [15]. We formally recommended superselective percutaneous transcatheter embolization. The mother was counseled extensively on the risk of permanent erectile dysfunction. However, at this point the mother desires continued observation with possible intervention before puberty.

There is a paucity of data on the long term outcomes of superselective embolization in the treatment of high flow priapism in the pediatric population. In one series by Cantasdemir et al., they reported their series of superselective embolization in seven children with high flow priapism. Of these 7 children, 6 only required one treatment while 1 required an additional treatment. At a median follow-up of one year there was complete resolution of priapism with no signs of erectile dysfunction [16]. However, to our knowledge this is the longest follow-up reported in the literature.

Surgical ligation of the internal pudendal or cavernosal arteries is another potential treatment option. However, it is an invasive procedure that carries a significant risk of permanent erectile dysfunction [17]; that is why we chose to recommend embolization as a first-line option.

## 4. Conclusion

High flow priapism is an extremely rare phenomenon in the pediatric population and is usually associated with perineal or genital trauma. We report the first case of high flow priapism caused by DPNB during a circumcision. The patient and family should be counseled on the different management options ranging from observation to surgical intervention and the associated risk of permanent erectile dysfunction with invasive management.
